# Topical Uses of Ergot

**Published:** 1880-03-20

**Authors:** 


### *TOPICAL USES OF ERGOT.*

In the American Journal of the Medical Sciences, July, 1879, Dr. Wm. B. Dabney mentions some local uses of ergot. He writes:

In cases of pterygium I have used it with decided benefit. A solution was applied three times a day, and the growth was checked thereby. In none of the cases where I have used it thus far has it exerted a curative action, but it is highly probable that if persisted in the bloodvessels supplying the pterygium would become so much contracted as to cause an actual diminution in the size of the growth.

In pharyngitis I have obtained excellent results from the application of a solution of Squibb's solid extract of ergot to the throat; indeed on other remedy has given anything like such satisfactory results in my hands. Just as in ophthalmia, the remedy seems to act much better in chronic than in acute cases. It is especially applicable when the blood-vessels of the pharynx are enlarged and tortuous, and when the secretion is not very great. In those cases where the mucous membane is thickened, it acts much more slowly, and in acute cases it possesses no advantages over other remedies. In affections of the pharrynx, and in other cases to be mentioned hereafter, a combination of ergotine with tincture of iodine, as in the following formula, is especially efficacious :

℞ Ergotine.......................................... gr. xx.

Tinc. iodine..........................................fl ʒj.

Glycerine..................................to make fl ℥j. M.

To be applied to the pharynx freely, twice a day, with a camel's hair brush.

In hypertrophy of the tonsils, which is so often an accompaniment of chronic pharyngitis, the same solution applied to the glands two or three times a day gives excellent results.

It is probable that nasal catarrh would be benefitted by ergot, locally applied. The great trouble in these cases has been that remedies applied with the nasal douche have remained in contact with the congested Schneiderian membrane too short a time to do any good. About two years ago Dr. George Catti proposed the use of gelatine bougies, which were to be inserted through the anterior nares, and then allowed to soften and flow out by the posterior nares. These bougies could be medicated with any agent which it was thought desirable to use, and in a note appended to the translation of Catti's paper in the Virginia Medical Monthly I suggested the use of ergot in this way. I have never tried the bougies myself, however. In one case of catarrh, when the inflammation was seated near the posterior nares, I applied a solution of ergot and iodine by means of the post-nasal syringe, but the result of the treatment is not known. A solution of ergot in glycerine may also be applied to the nasal mucous membrane by means of a camel's-hair pencil, but I cannot say that I have had satisfactory results from any mode of application which I have tried thus far. If the medicine be applied to the Schneiderian membrane in any way, the iodine should not be added to the mixture at all, or else only in very small quantity.

It is unnecessary to say anything as to the use of this agent in hemorrhoids, as it is mentioned now in nearly all the text-books on therapeutics, and is in common use.

It seems almost needless, also, to say anything as to its use in metritis and endometritis. But, although it is mentioned now in nearly all the works on gynæcology, its value does not seem to be recognized by the majority of general practitioners.

It appears to be especially applicable in cervical metritis. The manner in which it should be applied depends on the season of the year and the temperature. When the weather is sufficiently cool suppositories are preferable, but in warm weather it is difficult to handle them and keep them from melting. The addition of extract of belladonna increases the efficacy of the ergot, and also tends to relieve any pain which may be present.

The following formula I have found serviceable :

℞ Ergotine (or solid extract of ergot)................ grs. xx.
Extract of belladonna .............................. grs. ij.
Cocoa butter.......... ................................ q. s. M.

Make into six suppositories, and insert into the vagina every night after using the hot douche.

In warm weather a solution of ergotine and extract of belladonna in glycerine and water may be used in place of the suppositories, as in the following formula :

℞ Ergotine (or Squibb's solid extr.)........................... ʒ ss.
Extract of belladonna............................................ grs. vj.
Water and glycerine.........................................aa f℥ iv. M.

A pledget of cotton is to be saturated with this solution, and inserted into the vagina at bed time after the hot douche. The cotton should, of course, be removed in the morning.

It has been proposed to paint a solution of ergot on the os and cervix with a camel's-hair pencil, and favorable reports of this mode of treatment have been published. So far as my own experience enables me to judge, those cases where there is a copious discharge of mucus or pus are much less amenable to treatment than others, and this is probably due to the fact that the medicine remains in contact with the diseased surface such a short time before it is washed off. And I would call attention just here to the advantages of glycerine over water as a vehicle when ergot is applied to mucous membranes where it is liable to be speedily washed off. The tenacious properties of glycerine keep the remedy longer in contact with the diseased surface, and in addition to this the glycerine itself is, as Dr. Marion Sims long ago pointed out, of decided value in reducing some of these chronic inflammatory engorgements.—*H. Y. Comp. of Med. Science.*

